# Single Cell Transcriptomes of *In Vitro* Bradyzoite Infected Cells Reveals *Toxoplasma gondii* Stage Dependent Host Cell Alterations

**DOI:** 10.3389/fcimb.2022.848693

**Published:** 2022-03-14

**Authors:** Tatsuki Sugi, Tadakimi Tomita, Taishi Kidaka, Naoko Kawai, Kyoko Hayashida, Louis M. Weiss, Junya Yamagishi

**Affiliations:** ^1^ Division of Collaboration and Education, International Institute for Zoonosis Control, Hokkaido University, Sapporo, Japan; ^2^ Department of Pathology, Albert Einstein College of Medicine, Bronx, NY, United States; ^3^ International Collaboration Unit, International Institute for Zoonosis Control, Hokkaido University, Sapporo, Japan; ^4^ Department of Medicine (Infectious Diseases), Albert Einstein College of Medicine, Bronx, NY, United States

**Keywords:** *Toxoplasma gondii*, scRNA-Seq, host–pathogen interaction, chronic infection, bradyzoite, single cell transcriptome

## Abstract

*Toxoplasma gondii* bradyzoites establish chronic infections within their host cells. Recent studies have demonstrated that several parasite effector proteins are translocated to host cells during the bradyzoite stage of chronic infection. To understand the interaction between host cells and bradyzoites at the transcriptomic landscape level, we utilized single-cell RNA-sequencing (scRNA-Seq) to characterize the bradyzoite-induced host cell response. Distinct gene expression profiles were observed in infected host, cells with low parasite mapped reads, and mock (non-exposed) control cells. Gene set enrichment analysis showed that c-Myc and NF-κB signaling and energy metabolic pathways were upregulated by infection. Type I and II interferon response pathways were upregulated in cells with low parasite mapped reads compared to the non-exposed host control cells, and this upregulation effect was reversed in infected cells. Differences were observed in the host cells depending on the differentiation status of the parasites, as determined by BAG1 and SAG1 expression. NF-κB, inflammatory response pathways, and IFN-γ response pathways were downregulated in host cells containing *T. gondii* ^BAG1+/SAG1−^, whereas this downregulation effect was reversed in case of *T. gondii* ^BAG1−/SAG1+^. We also identified two distinct host cell subsets that contained *T. gondii* ^BAG1+/SAG1−^, one of which displayed distinct transcriptomes with upregulated c-Myc expression. Overall, these data clearly demonstrate that host cell transcriptional alteration by bradyzoite infection is different from that of tachyzoite infection, indicating fine-tuning of the host immune response.

## Introduction

*Toxoplasma gondii* is an apicomplexan pathogen capable of reactivating encephalitis in immune-compromised hosts (McLeod et al., 2014). During primary (i.e., initial) infection, *T. gondii* differentiates from rapidly growing disseminating tachyzoites into slowly replicating bradyzoites, which are in tissue cysts (a modified parasitophorous vacuole) formed in neuronal or muscular cells, resulting in latent (i.e., chronic) infection. These tissue cysts are the source of parasites that cause encephalitis in immune-compromised patients (McLeod et al., 2014). Moreover, such cysts in meat production animals serve as a major source for primary human infection (Cook et al., 2000). It has been observed that during the prolonged period of chronic infection, host immune system can clear some tissue cysts (Suzuki, 2020); however, the majority of them do not trigger a direct immune response or inflammatory cell accumulation (Ferguson et al., 1994). The exact mechanisms by which bradyzoite-infected host cells escape the host immune response are unknown.

Host transcriptional alteration is a key mechanism used by many pathogens to establish a stable parasitic niche. Various *T. gondii* effectors are known to cause host transcription alterations at acute stage of tachyzoite infection. These include ROP16, GRA6, GRA15, and GRA16 which activate STAT3 signaling (Yamamoto et al., 2009), NFAT4 (Ma et al., 2014), NF-κB (Rosowski et al., 2011; Ihara et al., 2020), and p53/c-Myc pathways (Bougdour et al., 2013; Panas and Boothroyd, 2020), respectively. Additionally, GRA18 activates the anti-inflammatory response through β-catenin upregulation (He et al., 2018), GRA24 activates p38 MAPK signaling (Braun et al., 2013), TgIST (Gay et al., 2016; Matta et al., 2019) /TgNSM (Rosenberg and Sibley, 2021) suppresses the IFN response pathway, and TEEGR/HCE1 (Braun et al., 2019; Panas et al., 2019) and MAG1 (Tomita et al., 2021) counteract GRA15 dependent NF-κB signaling *via* E2F pathway activation or suppressing the inflammasome activity.

Among these effectors, TgIST and TgNSM are known to cross the cyst wall, translocate to host cell nuclei, and suppress the IFN response in host cells containing bradyzoites (Mayoral et al., 2020; Rosenberg and Sibley, 2021; Seizova et al., 2021). Additionally, TgNSM has been recently reported to play a role in the suppression of IFN-γ-related necroptosis in bradyzoite-containing host cells (Rosenberg and Sibley, 2021). On the contrary, several effectors with activity in tachyzoite-containing host cells namely, GRA16 and GRA24 are not secreted across the cyst wall (Krishnamurthy and Saeij, 2018). These observations indicate that distinct host alterations occur in the bradyzoite-infected host cells.

In addition to the characterization of specific parasite effectors in bradyzoite-infected host cells, transcriptome analysis of these cells can reveal the underlying bradyzoite–host interactions. Currently, the most widely used bradyzoite induction conditions involve a low infection rate to obtain well-differentiated bradyzoites (Fouts and Boothroyd, 2007). Previous transcriptome analysis of human fibroblast cells infected *in vitro* with *T. gondii* under bradyzoite induction conditions demonstrated subtle transcriptional changes in host cells (Fouts and Boothroyd, 2007). It is difficult to clearly detect transcriptome changes caused by bradyzoite infection, as the cells in the monolayer included bystander-uninfected cells, cells with tachyzoites, and cells with bradyzoites. More recently, Seizova et al. characterized the host cell transcriptome in bradyzoite-infected cells by analyzing cells enriched by bradyzoite-specific BAG1 promoter activity using FACS (Seizova et al., 2021). They found that infected, bystander-uninfected, and mock-treated cells have distinct transcriptomes. In particular, interferon response pathways are upregulated in bystander cells, while these are downregulated in infected cells, depending on MYR1 translocon and TgIST (Seizova et al., 2021). This study suggested that solving the problem of heterogeneity in the *in vitro* bradyzoite induction culture (i.e., mixture of bystander-uninfected, tachyzoite-infected, and bradyzoite-infected cells) is critical for studying the effect of bradyzoites on the host cells. As an alternative approach to deepen the understanding of host transcriptome alteration by bradyzoite infection, we planned to solve the problem of heterogeneity in the bradyzoite induction culture using single-cell level transcriptome analysis, which can reveal the heterogeneity among bradyzoite-infected cells as well.

Using single-cell RNA sequencing (scRNA-Seq), we obtained host cells and parasite transcriptome for each cell successfully. Using host/parasite-paired single-cell transcriptome data, we determined the host cell alterations caused by *T. gondii* in an *in vitro* bradyzoite culture condition in infection-dependent and stage differentiation-dependent manner. These observations were further validated by biologically independent and different-scale scRNA-Seq datasets with different bradyzoite infection rates to obtain a rigorous conclusion independent of the infection rates.

## Materials and Methods

### Cell Culture

Human foreskin fibroblasts (HFFs) were passaged serially with Dulbecco’s modified Eagle’s medium (DMEM) (Thermo Fisher) supplemented with 10% FBS (no antibiotics added) (10% FBS DMEM). *T. gondii* ME49 parasites were passaged and propagated using confluent HFF cells in DMEM supplemented with 10% FBS as described previously (Tu et al., 2019).

### Bradyzoite Induction

Extracellular parasites were obtained by rupturing infected host cells with a 27 G needle three times, followed by filtration through a 5 µm pore PVDF filter (Millipore). A total of 2.0 × 10^5^ parasites/well were inoculated into confluent HFF cells in a 6-well plate and incubated for 2 h at 37°C and 5% CO_2_. This was followed by washing twice with phosphate buffered saline (PBS) to remove uninvaded parasites. The medium was then replaced with RPMI 1640 (Thermo Fisher) supplemented with 1% FBS, 25 mM HEPES, and pH 8.1 (bradyzoite medium) (Abdelbaset et al., 2017). Infected cells were incubated in a humid incubator at 37°C for six days without CO_2_. On days 2 and 4 post-infection, the medium was replaced with fresh bradyzoite medium. On day 6 post-infection, cells were subjected to scRNA-Seq analysis. The HFF cell controls were prepared in parallel with the infected cells in same manner, except that the parasites were not inoculated. For the large cell number dataset, bradyzoite induction was performed with a difference of 6 h in the invasion window, instead of 2 h to increase the infection rate.

### Single-Cell RNA Sequencing

Cells were rinsed twice with 2 ml of cold PBS and incubated with 0.5 ml TrypLE Express (Thermo Fisher) at room temperature for 5 min. Trypsin treatment was quenched with the addition of 0.5 ml of cold DMEM with 10% FBS and detached cells were centrifuged at 200×*g* for 5 min at 4°C. The cell pellets were resuspended in cold PBS by pipetting 20 times to prepare a single-cell suspension. A total of 2.0 × 10^4^ cells were loaded onto the BD Rhapsody platform to create the sequence library for scRNA-Seq with a unique cell barcode corresponding to a single-cell transcriptome according to the instructions of the manufacturer. After capturing single-cell transcriptome for each mRNA capture bead, one out of five total mRNA capture beads was used for further scRNA-seq library preparation to obtain 4,000 cells for analysis. Due to loss of some cells, a library of total 1,534 cells was obtained. Each library was then sequenced with HiSeq X to generate approximately 240 million read pairs (~480 million reads) per sample. The remaining single-cell suspension was attached to a glass slide with cytospin and stained with *Dolichos biflorus* agglutinin conjugated with fluorescein isothiocyanate (DBA-FITC) for cyst wall (Vector Lab), anti-Toxoplasma polyclonal rabbit antibody (BioRad) for total parasites, and DAPI for cell nuclei as described by Tu et al. (2019) to estimate the infection ratio. For the large cell number dataset, approximately 50,000 cells were processed with the Rhapsody scRNA-Seq platform and one out of five total mRNA capture beads corresponding to approximately 10,000 cells was used for the scRNA-Seq library preparation. Because this large cell number dataset had different cell numbers, resulting in different sequence depths from non-exposed mock control cells, the comparison between these two datasets was not performed. Sequence data were made accessible at DDBJ Sequence Read Archive under accession numbers DRR318618, DRR330607, and DRR318701 for bradyzoite-infected HFF cells (small cell number deep RNAseq dataset), bradyzoite-infected HFF cells (large cell number sparse RNAseq dataset), and mock-infected HFF cells, respectively.

### Quality Filtering, Cell Assignment, Sequence Alignment, and Gene Count

Fastq files for paired-end reads were processed using BD Rhapsody™ genome analysis pipeline version 1.9.1 according to the instructions of the manufacturer to obtain a gene expression table of the unique molecular counts for each gene in individual cell. The human reference genome and annotation files (GRCh38 of release-102) were retrieved from the Ensemble website (Yates et al., 2020) (https://www.ensembl.org), and the *T. gondii* ME49 strain genome and annotation files were retrieved from ToxoDB (https://toxodb.org) (Harb and Roos, 2020) release 50.

### Data Preprocessing

Cells of good quality with mitochondrial gene percentage less than 15% were filtered. For every single cell, the count per million (CPM) reads mapped to each host gene was calculated. Log_10_ (CPM + 1) was used as log-normalized data. For parasite-mapped reads, the CPM was calculated with only the reads mapped to the parasite transcriptome. The bulk RNA-Seq for each dataset was simulated by totaling the gene expression count from the whole cells in each dataset. The CPM reads mapped to the host cell were calculated for each gene in the bulk RNA-Seq dataset and are summarized in **Dataset S1, Sheet 1**.

### Clustering and Dimensional Reduction

Based on the host gene expression profiles, clustering of cells was performed using R packages, scatters, (McCarthy et al., 2017), and scran (Lun et al., 2016) for cells from bradyzoite-exposed culture and mock control culture. The sequence read depth of the bradyzoite-exposed culture was adjusted through random sampling with a rate of 0.32 to get equivalent median molecule RNA-seq depth per cell.

### Differentially Expressed Gene (DEG) Analysis

DEGs were estimated using a zero-inflated negative binomial model. For host cell DEG analysis, the ZINB-WaVE (Risso et al., 2018) weight estimation combined with the DESeq2 (Love et al., 2014) pipeline was used. For parasite DEG analysis, ZINB-WaVE weight estimation was combined with edgeR (Robinson et al., 2010) pipeline for the robust detection, even if the depth of the parasite-mapped transcriptome data was low. The table for DEGs between host cells infected with each differentiated parasite and cells with low parasite mapped reads is provided in **Dataset S1, Sheet 2**.

### Gene Set Enrichment Analysis (GSEA)

GSEA (Subramanian et al., 2005) was performed using fGSEA package (Korotkevich et al., 2016). All genes were used to prepare a ranked gene list by calculating the rank of each gene as described previously (Van den Berge et al., 2018). For the human gene set, we used the hallmark gene set from the MSigDB (Liberzon et al., 2015). For the *T. gondii* gene set, we used curated KEGG pathways and bradyzoite-related genes (Croken et al., 2014). *T. gondii* genes specifically expressed in extracellular parasites were retrieved from ToxoDB with an adjusted *p*-value <0.05 and log_2_ fold change over two, using the transcriptome dataset of intracellular and extracellular parasites (Hassan et al., 2017).

### Host Cell Cycle Phase Estimation

Host cell cycle estimation was performed with R package Seurat version 4 (Hao et al., 2021) using cell cycle gene set (Tirosh et al., 2016) with default parameter settings in Seurat package.

### Statistical Analyses

For DEGs, we used the DESeq2 package to perform likelihood ratio tests (Love et al., 2014). For GSEA, we used fGSEA to calculate the adjusted *p*-value with an adaptive multilevel split Monte Carlo scheme (Korotkevich et al., 2016). One-way ANOVA test with *post-hoc* Tukey’s HSD test was used for multiple group comparisons, whereas the Student’s t-test was used for comparison of two groups.

## Results

### Single Cell RNA-Sequencing Allowed *T. gondii-*Infected Host Cells to be Successfully Assigned Based on Both Infection and Parasite Differentiation Status

**Figure 1:**
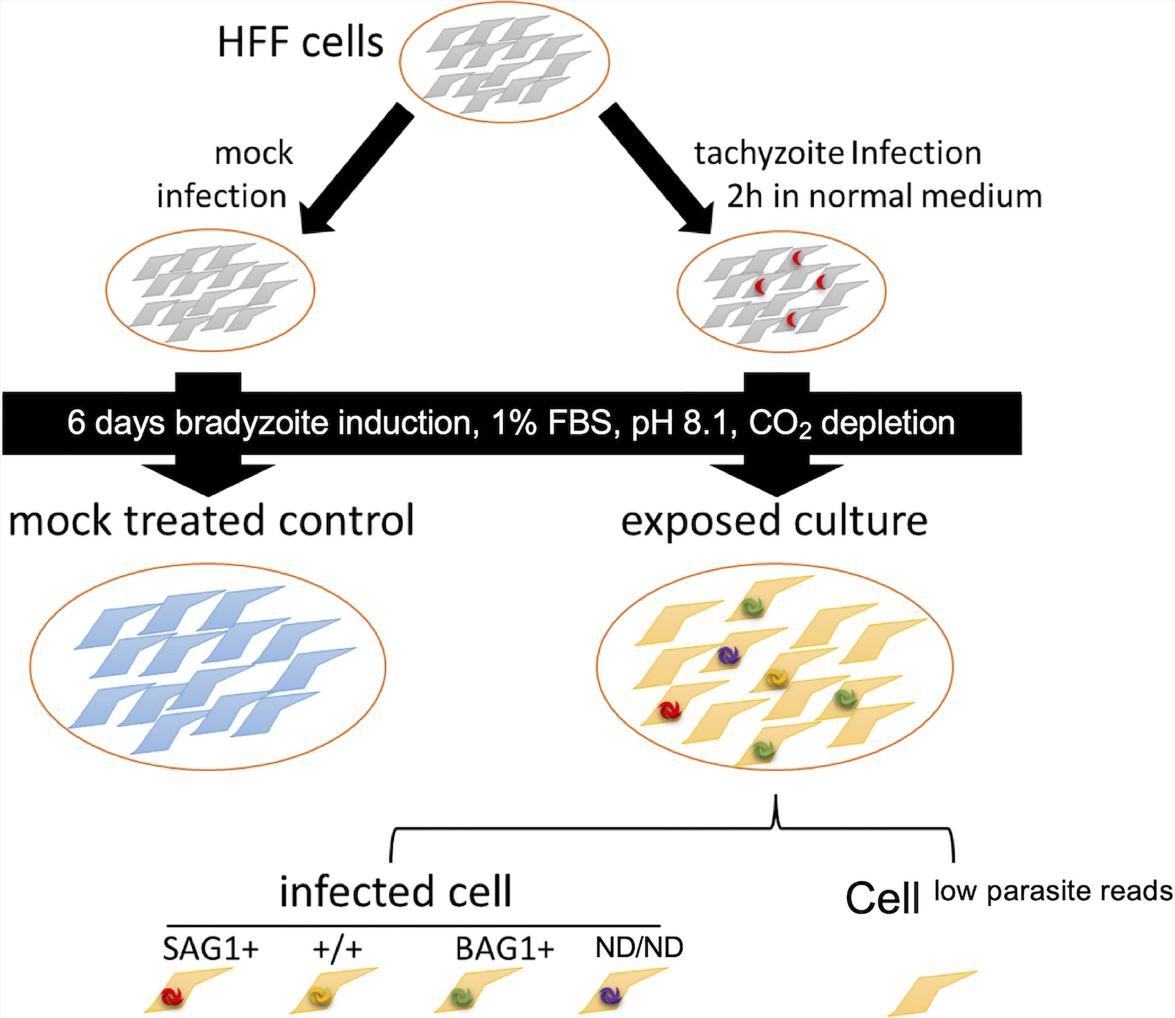
Schematic diagram of the experimental design. For transcriptome analysis of parasite-infected cells, we prepared bradyzoite-infected cells (exposed culture) by inoculating HFF cells with tachyzoites, followed by the induction of bradyzoite differentiation for 6 days in bradyzoite induction medium without CO_2_ addition. As a control, HFFs without parasite inoculation were incubated under the same conditions for 6 days (mock-infected treated control). In the exposed culture, the cells were assigned to each category based on the mRNA molecules detected. Parasite-mapped mRNA molecules (parasite load) were used to infer the infection status, and the cells were divided into infected cells and others (cells^low parasite reads^). Infected cells were further assigned to different subsets based on the presence of the canonical tachyzoite marker SAG1 and bradyzoite marker BAG1 (SAG1+: cells with *T. gondii* ^SAG1+/BAG1−^, +/+: cells with both BAG1 and SAG1 markers were detected, BAG1+: cells with *T. gondii* ^SAG1−/BAG1+^, ND/ND: cells with *T. gondii*, but both SAG1 and BAG1 markers were below the detection limit).

HFF cells infected with *in vitro* differentiated bradyzoites (exposed culture) (**Figure 1**) were analyzed using scRNA-Seq. Single-cell transcriptome data from 1,534 cells with a median number of 27,430 mRNA molecules per cell were obtained. The remaining material from the single cell suspension used for scRNA-Seq was characterized by IFA, which demonstrated that 5.1% of host cells were infected with at least two parasites (**Supplementary Figure S1A**).

**Figure 2:**
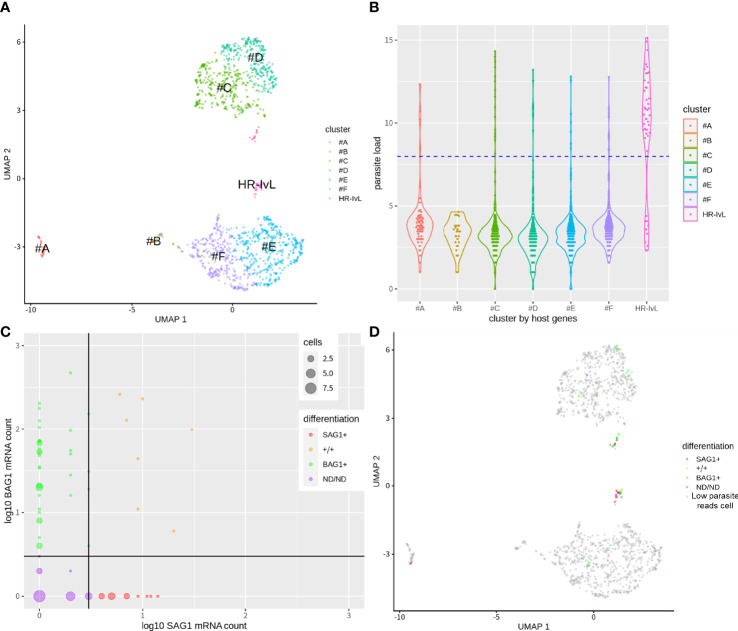
Clustering of host cells in bradyzoite-induced culture with single cell transcriptome. **(A)** Single cell level transcriptome of parasite-exposed host cells in bradyzoite induction condition. Host cell transcriptome was used for the clustering of host cells. HR-IvL: a cluster with a high ratio of infected cells vs. cells^low parasite reads^. **(B)** Log_2_ transformed mRNA molecule counts mapped to parasites were plotted to indicate parasite load per cell for each cluster. By counting the number of infected cells using immunofluorescence analysis, infection rate was estimated as 5.1%. The top 5.1% cells in parasite-mapped mRNA counts were assigned as infected cells. Blue dots line shows the cut-off level to separate putative infected and cells^low parasite reads^. **(C)** Infected cells were further plotted for the expression of canonical tachyzoite marker SAG1 and bradyzoite marker BAG1. Log_10_ values of mRNA count + 1 for each gene are shown. The cut-off line of raw mRNA count number above three was used to assign each cell as positive for SAG1/BAG1. A total of 78 putative infected cells were divided into 39 BAG1+, 11 SAG1+, 7 +/+, and 21 ND/ND cells. Size of dots show the cell number which shares the same mRNA count number. **(D)** Parasite differentiation category are overlayed with host cell clustering.

We then identified the host cell subsets using the host cell gene expression profiles and demonstrated that the cells were clustered into several groups on UMAP plot (**Figure 2A**). Host cells were clustered partially based on their cell cycle phase (G2M for clusters #A and #B, **Supplementary Figure S1B**) and intrinsic heterogeneity (clusters #C, #D, and #E, #F). These patterns were also observed in non-parasite-exposed mock control cells (**Supplementary Figure S1C**). Next, we assigned each cell type based on their putative infection status. In our scRNA-Seq dataset, putative bystander-uninfected cells also contained a few parasitic mRNA reads. A possible reason could be the spillover of the mRNA molecules during cell rupture and mRNA capture on each cell barcode bead in the nano-well format which was not separated completely. To estimate the infection status from the scRNA-Seq dataset, we utilized the mRNA mapped to the *T. gondii* transcriptome as an indicator of parasite load. The top 5.1% cells with the highest parasite loads were assigned as putative infected cells, based on the infection rate estimated by IFA. This was achieved by counting the cells with at least two parasites in order to exclude the cells with a single parasite that was liberated from the parasitophorous vacuole during the preparation of the single-cell suspension. Thus, infected cells with large parasitophorous vacuoles are more likely to be assigned as putative infected cells, whereas cells with few parasites could be misclassified as putative bystander-uninfected cells. We call these cells below the infection threshold (**Figure 2B**) as “cells with low parasite mapped reads (cells^low parasite reads^)” rather than “bystander-uninfected cells” to describe appropriately. This possible contamination of infected cells into putative bystander-uninfected cell subset (cells^low parasite reads^) could decrease the sensitivity of DEG detection caused by infection. By comparing the clusters identified in **Figure 2A** and the infection status of the cells in **Figure 2B**, we identified one cluster with a high ratio of infected cells vs cells^low parasite reads^ (HR-IvL) (35 infected cells vs. 10 cells^low parasite reads^) (**Figure 2B** and **Table S1A**). We further divided the infected cells according to their parasite differentiation status. For this purpose, the expression levels of the tachyzoite marker SAG1 and bradyzoite marker BAG1 were analyzed for each infected cell (**Figure 2C**). SAG1+/− and BAG1+/− were assigned using a threshold of mRNA count >2 for each infected cell. A negative mark during classification means that the expression level of marker mRNA was less than the threshold. As a result, infected cells were classified into the following four subsets: host cells with tachyzoite-like *T. gondii^SAG1^* ^+/BAG1−^ (SAG1+; n = 11), host cells with bradyzoite-like *T. gondii* ^SAG1−/BAG1+^ (BAG1+; n = 39), host cells with both SAG1 and BAG1 markers (+/+; n = 7), and host cells without SAG1 and BAG1 (ND/ND [SAG1 Not Detected/BAG1 Not detected]; n = 21). A comparison of these differentiation-based assignments and host clustering demonstrated that all four infected subsets were found in the cluster “HR-IvL” (**Figure 2D** and **Table S1A**).

To validate our scRNA-Seq pipeline with more cell numbers and to check whether the detected changes were susceptible to bradyzoite induction, scRNA-Seq was performed with biologically independent bradyzoite samples with large cell numbers (**Large dataset**). For this dataset, we used an invasion window of 6 h instead of 2 h to increase the number of infected cells for analysis. A total of 12,479 cells were passed through an intact cell filter with around 20% infection rate. Although the infection rates were different for the small cell number deeply sequenced dataset (**Deep dataset**) and large cell number scRNA-seq dataset (**Large dataset**), unsupervised cell clustering revealed one cluster “HR-IvL,” which had a high ratio of infected cells vs. cells^low parasite reads^ (363 infected cells vs. 45 cells^low parasite reads^) in the Large dataset (**Supplementary Figures S2A, B** and **Table S1B**). Owing to the large number of cells and low RNA-Seq depth for every single cell, few SAG1 and BAG1 markers were detected per cell (**Supplementary Figure S2C**). In the large dataset, we classified infected cells into four subsets according to SAG1 and BAG1 marker expression levels (BAG1+; n = 1,604, SAG1+; n = 5, +/+; n = 3, and ND/ND; n = 877) (**Supplementary Figures S2C, D**, and **Table S1B**).

Bulk RNA-seq data (sum of the number of mRNA molecules obtained from all cells in the scRNA-seq dataset) of the Deep and Large datasets were analyzed to determine the differences in bradyzoite induction strength. As shown in **Table S1C**, both datasets showed high bradyzoite gene expression levels; however, the Large dataset exhibited better bradyzoite differentiation than the Deep dataset. This could be the possible reason for the difference observed in the SAG1+ to BAG1+ ratio between the Deep and Large datasets (39 BAG1+ vs 11 SAG1+ in the Deep dataset and 1,604 BAG1+ vs 5 SAG1+ in the Large dataset). However, we cannot exclude the possibility that sequence depth per cell may affect the detection levels and relatively lower expression of SAG1 was probably below the detection limit in the Large dataset. By merging the two datasets for eliminating the batch effect derived from the cell number and RNA-Seq depth differences, we identified that both datasets had a cluster with high proportion of infected cells (**Supplementary Figures S2E, F**, cluster 7). Collectively, these results suggest that under *in vitro* bradyzoite induction culture conditions, host cell transcriptome was altered as a result of infection with parasites in various differentiation stages, independent of the induction conditions. Further analyses were conducted using the Deep dataset because we intended to use deep RNA-seq data for reliable analysis.

### Host Transcriptome Alteration Caused by Infection Correlated With the Cluster “HR-IvL”

**Figure 3:**
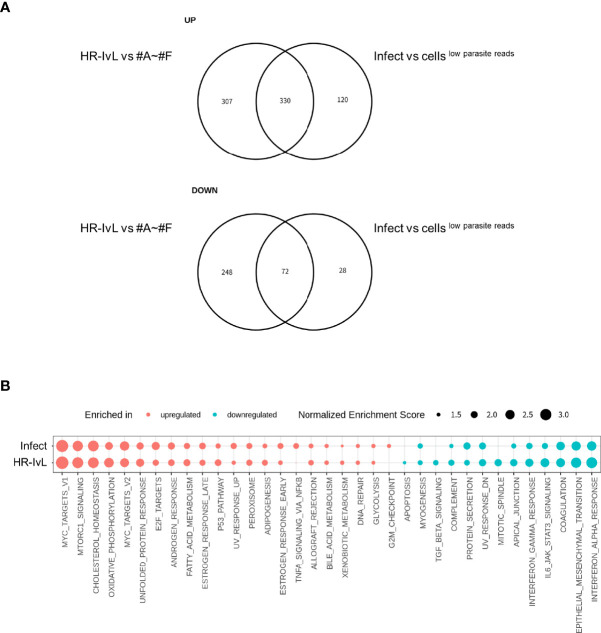
Altered host gene expression in infected cells is correlated with that detected in the host cell subset “HR-IvL”. **(A)** Genes with log_2_ fold change over 0.5 and Benjamini–Hochberg adjusted *p-value* (False Discovery Rate: FDR) less than 0.05 were flagged as differentially expressed genes (DEGs). Upregulated (top panel) or downregulated (bottom panel) DEGs in infected cells vs. cells^low parasite reads^ were compared with those in “HR-IvL” vs. “#A–#F” clusters. **(B)** Normalized enrichment scores for gene set enrichment analysis (GSEA) are shown. Enriched pathways for upregulated genes are shown in red, and those for downregulated genes are shown in blue. Enriched pathways with FDR less than 0.05 in either infected cells vs. cells^low parasite reads^ or HR-IvL vs #A–#F are shown. Pathways with FDR < 0.05 are shown with solid circles. “Infect” and “HR-IvL” indicate infected cells vs. cells^low parasite reads^ and HR-IvL vs. #A–#F. respectively.

As described in previous section, unsupervised clustering shows that the proportion of the infected cells clustered separately from the other cells^low parasite reads^ (i.e., infected cells in HR-IvL) was low (35 in HR-IvL out of 78 total infected cells and 363 in HR-IvL out of 2,489 total infected cells, in the Deep and Large datasets, respectively; **Tables S1A, B**). This suggests that the host alteration caused by parasite infection, if any, was not as large as the intrinsic diversity of the host cell transcriptome at the single-cell level. To test if host alteration in the “HR-IvL” correlated with that in the infected cells, DEGs were compared. A total of 330 (out of 450) upregulated and 72 (out of 100) downregulated DEGs in infected cells vs. cells^low parasite reads^ were identified in the “HR-IvL” vs other clusters (**Figure 3A**). We conducted GSEA using the human hallmark pathways (Liberzon et al., 2015) (**Figure 3B**) and found that c-Myc target, oxidative phosphorylation, cholesterol homeostasis, fatty acid metabolism, E2F target, and p53 pathways were enriched for the upregulated genes in the infected cells and in the “HR-IvL”. Immune responses including the IFN-α/γ response pathways were enriched for the downregulated genes in the infected cells and in the “HR-IvL” cluster. These results suggest that clustering without any prior knowledge can detect the host cell subset affected by infection. As shown in **Table S1A**, the infected cell population in “HR-IvL” had all the four subsets, i.e., SAG1+, BAG1+, ND/ND, and +/+. This indicates that the infection under the *in vitro* bradyzoite culture conditions had an overall impact on the host transcription and all the differentiated parasites were clustered together in “HR-IvL.” To investigate the differences in host alteration in the infected cells by each differentiation subset, each subset was further analyzed.

### Infected Cells and Cells^low parasite reads^ Exhibited Altered Gene Expression Compared to Mock-Infected HFF Cells

**Figure 4:**
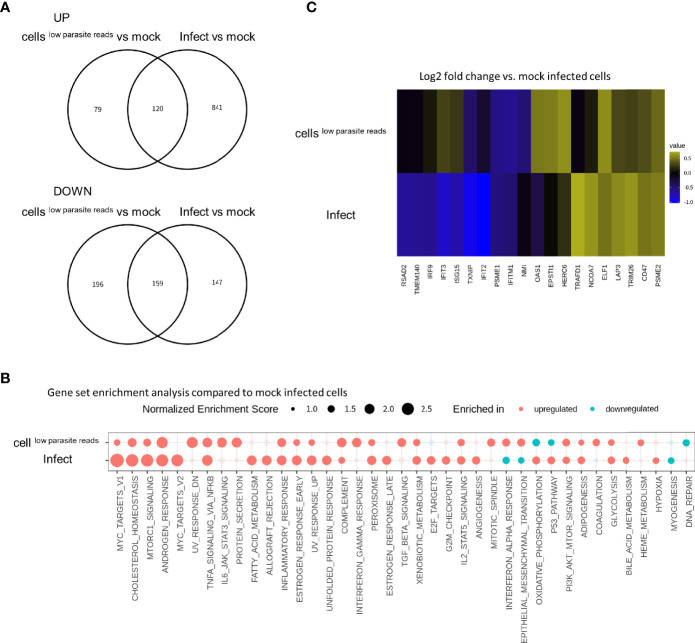
IFN-α response pathway is upregulated in cells^low parasite reads^ and this effect is reverted in infected cells. **(A)** DEGs detected in cells^low parasite reads^ vs. mock control (labeled as cells^low parasite reads^) and those detected in infected cells vs mock control (labeled as **infect**) were compared in Venn diagrams. Log_2_ fold change of 0.5 and FDR of 0.05 were used to estimate DEGs. Number in the circle indicates number of DEGs. **(B)** Normalized enrichment scores for gene set enrichment analysis (GSEA) are shown. Enriched pathways for upregulated genes are shown in red and those for downregulated genes are shown in blue. Enriched pathways with FDR less than 0.05 in at least one of the comparisons are shown. Pathways with FDR < 0.05 are shown with solid circles. **(C)** Heatmap visualization of genes in IFN-α response pathway. Log_2_ fold change values for each gene are shown with pseudo colors. Only genes with absolute values of Log_2_ fold change over 0.5 in at least one of the comparisons are shown.

Next, we examined whether cells^low parasite reads^ and/or infected cells exhibited DEGs compared to “mock-infected” cells. For this purpose, control HFF cells were prepared by incubation for 6 days under bradyzoite induction culture conditions, as was done for the infected cell culture (**Figure 1**). A substantial number of genes were uncommon among the DEGs detected in the cells^low parasite reads^ or infected cells compared to the unexposed or “mock-infected” control cells (**Figure 4A**). GSEA demonstrated that some of the pathways in cells^low parasite reads^ and infected cells were reverse regulated, namely, the IFN-α response, oxidative phosphorylation, and epithelial and mesenchymal transition pathways (**Figure 4B**). Further analysis using a heatmap of the IFN-α response pathway genes (**Figure 4C**) also showed that some of the upregulated genes in cells^low parasite reads^, namely, *IRF9*, *IFIT3*, *ISG15*, *OAS1*, *EPSTI1*, and *HERC6*, were downregulated in the infected cells (**Figure 4C** and **Table S2A**). This result indicates that not only the infected cells but also the cells^low parasite reads^ had an altered host gene expression.

### Bradyzoite-Induced Changes in the Host Cell Transcriptome

**Figure 5:**
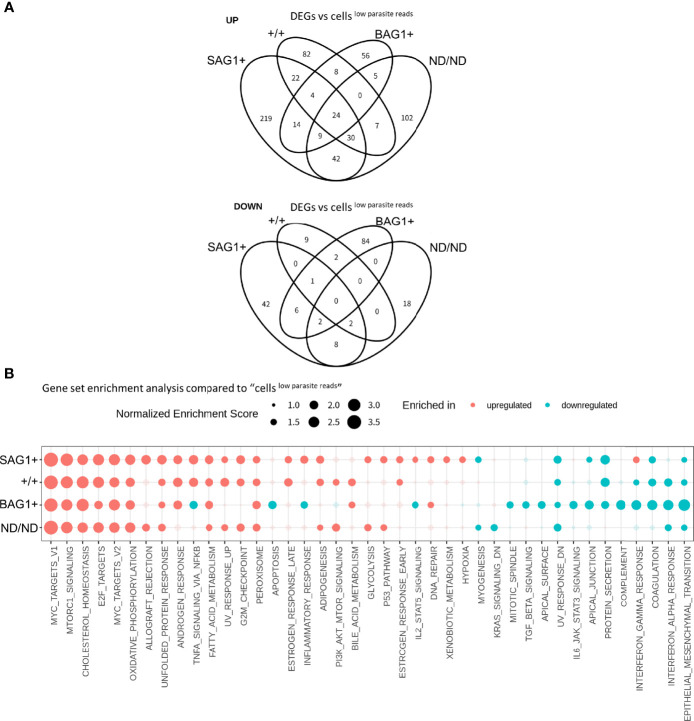
Host transcriptome alteration is dependent on the parasite differentiation status. **(A)** DEGs detected in host cell subsets (BAG1+, SAG1+, +/+, ND/ND respectively) vs. cells^low parasite reads^ were compared in Venn diagrams. Log_2_ fold change of 0.5 and FDR of 0.05 were used to estimate DEGs. Number in the circle indicates number of DEGs. **(B)** Normalized enrichment score for gene set enrichment analysis (GSEA) are shown. Enriched pathways for upregulated genes are shown in red and those for downregulated genes are shown in blue. Enriched pathways with FDR less than 0.05 in at least one of the comparisons are shown. Pathways with FDR < 0.05 are shown with solid circles.

To characterize the host cell transcription alterations in the four subsets of infected cells (SAG1+, +/+, BAG1+, ND/ND) (**Figure 2C**), we first examined whether these four subsets represented canonical tachyzoite- and bradyzoite-infected cells. The parasitic mRNA molecule count per cell in these four subsets indicated that the parasite load differed among the four subsets (**Supplementary Figure S3A**). The median values of the number of detected parasite genes (row count >2) for SAG1+, +/+, BAG1+, ND/ND were 135, 980, 201, and 52, respectively. Low sequence depth in the parasitic transcriptome means that we cannot fully exclude the possibility of SAG1/BAG1 misclassification, especially in the “ND/ND cells,” which had the lowest number of parasites detected. As a single host cell contained multiple parasites, the +/+ signal could be a result of *T. gondii* expressing both genes or due to the presence of both tachyzoites and bradyzoites in the same host cell. Therefore, we assumed that ND/ND and +/+ cells consisted of cells infected by parasites in various differentiation stages that could not be detected or assigned by analyzing expression of SAG1 and BAG1 markers in the scRNA-Seq dataset. Nonetheless, GSEA using a *Toxoplasma* gene profile with these four subsets indicated that upregulated genes in the BAG1+ and SAG1+ subsets were enriched in bradyzoite and tachyzoite genes, respectively (**Supplementary Figure S3B**). The +/+ and ND/ND cells had intermediate enrichment scores between those of SAG1+ and BAG1+ with respect to tachyzoite/bradyzoite gene expression (**Supplementary Figure S3B**). These GSEA results suggest that the BAG1+ and SAG1+ subsets represent the canonical parasite differentiation status. With regard to host transcriptome alterations, all four subsets had different profiles, as shown in the host cell DEG results (**Figure 5A**). It should be noted that the +/+ subset has only seven cells, and analysis for this subset is not sufficiently robust. Notably, BAG1+ and SAG1+ subsets had significant enrichment of host gene pathways with differences in the NF-κB signaling pathways, inflammatory response, and IFN-γ response pathways (**Figure 5B** and **Data Set S1 Sheets 3–5**). To observe the differences in different bradyzoite induction conditions, the Large dataset was analyzed as described above and compared to cells^low parasite reads^ (N = 9,990). With limited number of cells in the SAG1+ and +/+ subsets of the Large dataset, the analyses for these subsets were not sufficiently robust. BAG1+ cells (N = 1,604) in the Large dataset also showed similar host alteration profiles, namely, upregulation of host c-Myc target and cholesterol homeostasis, and downregulation of interferon response pathways, inflammatory responses, and NF-κB signaling pathways, further validating the host transcriptional changes in bradyzoite-infected cells with large cell numbers (**Supplementary Figure S4**). Collectively, these data clearly demonstrate that host cells infected with *T. gondii* bradyzoites exhibit transcriptome alterations.

### Heterogeneity in Host Responses Can be Seen in Cells Infected With BAG1+ Subset

Host cell clustering combined with parasite infection and differentiation status assignment (**Figure 2D**, **Table S1A**) revealed that a substantial proportion of BAG1+ cells were clustered into #A–#F clusters. Compared to SAG1+ cells (8 cells in HR-IvL and 3 cells in #A–#F), the proportion of BAG1+ cells (16 in HR-IvL and 23 in #A–#F) suggests that cells infected with bradyzoites tend to have more subtle transcriptional changes. As described above, the BAG1+ subset has already been classified as cells infected with bradyzoite-like parasites. We hypothesized that cells with bradyzoite infection could have heterogeneity in host transcription alterations.

**Figure 6:**
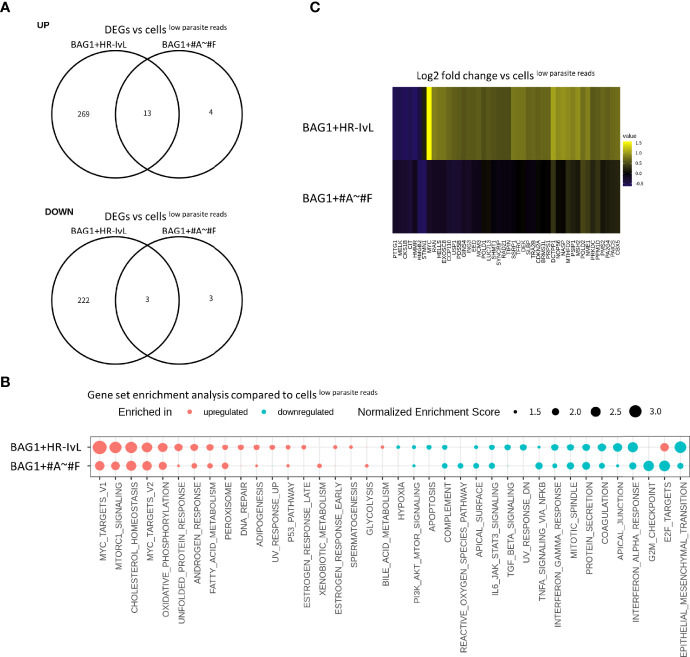
Heterogeneity exists in the cells containing *T. gondii* ^SAG1−/BAG1+^. **(A)** DEGs detected in BAG1+HR-IvL vs. cells^low parasite reads^ and BAG1+#A–#F vs. cells ^low parasite reads^ were compared in Venn diagrams. Log_2_ fold change of 0.5 and FDR of 0.05 were used to estimate DEGs. **(B)** Normalized enrichment scores for gene set enrichment analysis (GSEA) are shown. Pathways with FDR less than 0.05 in at least one of the comparisons are shown. Pathways with FDR <0.05 are shown as solid color circles. **(C)** Heatmap visualization of genes in E2F target pathway. Log_2_ fold change values for each gene are shown with pseudo color. Only genes with absolute values of log_2_ fold change over 0.5 in at least one of the comparisons are shown.

We found 16 BAG1+ cells in “HR-IvL” (BAG1+HR-IvL) and 23 BAG1+ cells in clusters #A–#F (BAG1+#A–#F). When these subsets were compared with cells^low parasite reads^, BAG1+#A–#F were found to be similar to the cells^low parasite reads^, with fewer DEGs than those detected in BAG1+HR-IvL (**Figure 6A**). Although the level of alteration was lower in BAG1+#A–#F, GSEA demonstrated that the direction of host transcription alterations was similar in these two subsets (**Figure 6B**). One notable difference was observed in the E2F target pathway. The E2F target pathway was upregulated in BAG1+HR-IvL, whereas it was downregulated in BAG1+#A–# F cluster. The heatmap analysis of genes in the E2F target pathway revealed that the most prominent difference was in the *c-Myc* expression level (**Figure 6C**, **Table S2B**). We attempted to validate this change at the protein level; however, we could not reliably detect this low-level expression of c-Myc in the cells under *in vitro* bradyzoite differentiation conditions (data not shown).

These heterogeneities in host cell response to infection encouraged us to examine the difference in parasitic gene expression in the BAG1+HR-IvL or BAG1+#A–#F subsets. The parasite load in the two subsets was not significantly different (**Supplementary Figure S5A**) and DEG analysis of parasites did not show significant differences with FDR <0.05 (**Data Set S1, Sheet 6** shows the DEGs with unadjusted p-value less than 0.05). GSEA using *T. gondii* genesets for KEGG pathway and a bradyzoite differentiation dataset (Croken et al., 2014) demonstrated that genesets “*in vivo* tachyzoite” and “KEGG pathway tgo03010 Ribosome” were enriched in the highly expressed genes in BAG1+HR-IvL (**Supplementary Figure S5B**), suggesting relatively active protein production in the parasites. Canonical bradyzoite markers showed similar expression levels in BAG1+HR-IvL and BAG1+#A–#F (**Supplementary Figure S5C**).

We examined if these subsets were subjected to the re-invasion of bradyzoites that released from the cells during the 6 days bradyzoite induction period. Time-course analysis of the bradyzoite induction culture showed that the infection rate did not change drastically during days 2, 4, and 6. Time-dependent growth of parasitophorous vacuole size indicated that a few new parasitophorous vacuoles emerged due to re-invasion events (**Supplementary Figure S6**). Another possible explanation is that bystander-uninfected cells were accidentally analyzed with extracellular *T. gondii* in the same nano-well as for single-cell separation, resulting in the misclassification as infected cells. GSEA using “extracellular parasites” geneset did not detect significant enrichment, excluding the possibility that extracellular parasites drove this heterogeneity in host cell transcription (**Supplementary Figure S5B**). Thus, we identified that transcriptomic heterogeneity exists in host cells containing *T. gondii* ^SAG1−/BAG1+^, even under well-controlled *in vitro* bradyzoite culture conditions.

## Discussion

Host cell manipulations have been observed in infections with various intracellular pathogens, namely, viruses (Finlay and McFadden, 2006), bacteria (Bhavsar et al., 2007), and parasites (Leirião et al., 2004). Studies on host cell manipulation by *T. gondii* have focused on tachyzoite-infection and have identified several parasite-derived effector proteins associated with host transcriptomic changes. Previous work on host cell responses to bradyzoite-infection demonstrated very limited transcriptional changes (Fouts and Boothroyd, 2007) compared to those observed in response to tachyzoite-infection (Blader et al., 2001), perhaps due to the presence of both infected and bystander-uninfected cells in cell cultures. In the current study, we successfully separated these two cell populations using scRNA-Seq. Additionally, we separated the infected cells into different subsets based on the differentiation status of the parasites.

Our scRNA-Seq level infection assignment had a limitation that putative bystander-uninfected cells also had parasite mapped reads, possibly because of mRNA spill over during single cell transcriptome capture. Thus, we could not separate bystander-uninfected cells with the cells with a few parasites completely. This could decrease the sensitivity in the detection of DEGs caused by infection compared to the ideal comparison of infected cells vs “pure” bystander-uninfected cells. Therefore, DEGs we detected may still underestimate the DEGs caused by bradyzoite.

Interestingly, the comparison of cells^low parasite reads^ and infected cells with non-exposed mock control cells revealed that the INF-α/γ response gene pathways were upregulated in cells^low parasite reads^ and downregulated in infected cells. Due to significantly high number of bystander-uninfected cells in parasite-exposed culture, activation of IFN signaling in the bystander-uninfected cells probably accounted for the observations in the previous studies examining the transcriptome of infected host cells in culture. Consistent with this hypothesis, IFN-γ response genes, namely, *STAT1*, *STAT3*, *DDX58*, and *HIF1A*, were found to be upregulated in a previous microarray analysis of exposed vs. non-exposed bradyzoite cultures (Fouts and Boothroyd, 2007). The reduced IFN-α or IFN-γ response in *T. gondii-*infected host cells compared to non-exposed cells is probably due to TgIST (Gay et al., 2016; Matta et al., 2019) and TgNSM (Rosenberg and Sibley, 2021). Since bystander cells do not contain *T. gondii*, upregulation of IFN signaling probably results from paracrine effects of infected cells or parasitic protein exposure (without infecting cells). For example, Rastogi et al. have shown that in tachyzoite-infection, bystander-uninfected but rhoptry-injected cells, bystander-uninfected and uninjected cells showed an upregulated IFN-γ response (Rastogi et al., 2020).

Owing to detection limits, the SAG1/BAG1 classification may not be a complete representation of the differentiated parasite subpopulations; however, we observed a clear difference in the host cell transcription alterations among the four subsets defined in our study. For example, despite the different infection time spans for transcriptome analysis, the host cell alteration of the SAG1+ subset in our study (under the bradyzoite induction culture conditions) was similar to that detected in a previous scRNA-Seq of tachyzoite-infected host cells (Rastogi et al., 2020). These alterations include upregulated NF-κB, inflammatory response, IL6-JAK-STAT3 signaling, IFN-γ response, MTORC1 signaling, and E2F target pathways and high allograft rejection.

We observed *T. gondii* ^SAG1−/BAG1−^ parasites in a substantial number of infected cells (21 ND/ND cells of 78 total infected cells). The ND/ND subset could be due to a failure to detect canonical markers (SAG1 and BAG1) as low depth of the mRNA reads was mapped to parasites at the single-cell level. Host cells with *T. gondii* ^SAG1+/BAG1+^ (+/+ subset) and SAG1+ subsets showed similar host transcription alterations, especially in the pathways which were reverse-regulated in SAG1+ and BAG1+ parasites, namely, NF-κB signaling, apoptosis, inflammatory response, and IL2-STAT5 signaling. The +/+ subset could be the result of co-infection of tachyzoites and bradyzoites in the same host cell, which cannot be excluded by the scRNA-seq platform. The presence of this +/+ subset with intermediate host transcription alterations between BAG1+ and SAG1+ indicates that the use of a single BAG1 marker expression to classify bradyzoites will categorize these SAG1+/BAG1+ in the bradyzoite population and interfere with characterizing bradyzoite specific host alterations.

The host transcription alterations observed in the BAG1+ subset, namely, downregulated NF-κB, inflammatory response, IL2-STAT5 signaling, and IFN-γ response were different than those in the SAG1+ subset, suggesting that these alterations are stage-specific. Interestingly, the balance between the activation and suppression of NF-κB pathway and the inflammatory response is manipulated by parasites to achieve a “balanced” host response. In our present dataset, we observed that bradyzoite-containing host cells had their transcriptomes “balanced” towards an anti-inflammatory response, i.e., suppressed states of NF-κB signaling and IFN-α/γ response pathways (**Figure 5B**). Activation of NF-κB signaling is caused by GRA15 (Rosowski et al., 2011; Sangaré et al., 2019; Ihara et al., 2020; Mukhopadhyay et al., 2020), which localizes to the parasitophorous vacuole membrane. One possible mechanism for the downregulated NF-κB signaling pathway in the BAG1+ subset is the reduced expression of GRA15 during bradyzoite differentiation as observed by microarray analysis (A dataset “Bradyzoite differentiation 3-day time series,” deposited by Drs. Fritz, Buchholz, and Boothryod to ToxoDB (https://toxodb.org) (Harb and Roos, 2020)) and RNA-seq analysis of life cycle of the parasite, namely, *in vitro* tachyzoite and *in vivo* tissue cysts (Ramakrishnan et al., 2019). Another possibility is the suppression of GRA15 effects by TEEGR/HCE1 (Braun et al., 2019; Panas et al., 2019) and MAG1 (Tomita et al., 2021). MAG1 counteracts the GRA15-mediated inflammatory activation by modulating inflammasome activity to interfere with IL1β secretion; however, it does not inhibit NF-κB activation by itself (Tomita et al., 2021). The NF-κB pathway was altered in our dataset; thus, MAG1 was unlikely to be the main contributor to this change. Another effector, TEEGR/HCE1, suppresses the NF-κB activation *via* induction of the E2F pathway (Braun et al., 2019; Panas et al., 2019). In BAG1+ cells, the E2F target pathway was upregulated compared to the cells^low parasite reads^ (the level of upregulation was lower than that in SAG1+ cells). Together with non-activated NF-κB signaling, the bradyzoite-infected cells had suppressed IFN signaling in our dataset. Failure to inhibit IFN-γ signaling through TgIST-KO and TgNSM-KO causes IFN-γ-dependent cyst clearance *in vitro* (Rosenberg and Sibley, 2021; Seizova et al., 2021). This suggests that bradyzoites suppress IFN signaling to evade immune attacks. The importance of IFN-γ signaling suppression in cyst maintenance suggests that “balancing” the NF-κB/IFN axis to an anti-inflammatory response in bradyzoite-infected host cells is beneficial for the latent persistent infections. To validate the NF-κB signal alteration detected by scRNA-Seq, we tried to detect p65 translocation in infected and bystander-uninfected cells; however, under the *in vitro* bradyzoite induction culture conditions (6 days incubation in RPMI medium with pH 8.1), we did not observe significant translocation of p65 in the host cell nucleus even in the bystander cells.

Recently, Seizova et al. enriched the bradyzoite-infected cells by FACS using BAG1 promoter activity as a bradyzoite marker and characterized bradyzoite dependent host transcriptional changes (Seizova et al., 2021). They identified that some host gene pathways are differentially regulated in tachyzoite- and bradyzoite-infected cells, namely, bradyzoite-specific IFN perturbations (Seizova et al., 2021). Also, bradyzoites rely upon MYR1 dependent protein translocation for the suppression of host cell IFN-γ response (Seizova et al., 2021). When results of the present study were compared with these findings, the host transcription alterations in infected and bystander-uninfected cells (in our analysis we used cells^low parasite reads^ instead) were found to be consistent among both datasets. Bystander-uninfected cells in the dataset by Seizova et al., and cells^low parasite reads^ in our dataset served as ideal controls with the same culture conditions and cell preparation steps from cell harvesting to (sc)RNA-Seq library preparation as that of the infected cells. Therefore, the consistent results observed for infected vs bystander-uninfected cells/the cells^low parasite reads^ suggested that both studies exhibited similar trends of host transcription alteration caused by bradyzoite infection. We also validated the biological replicate data with different cell numbers for scRNA-Seq to obtain a robust dataset independent of the analysis scales. Consistent with the previous dataset (Seizova et al., 2021), our large dataset also indicated the same host transcription alteration trends when infected cells were compared to the cells^low parasite reads^. In the dataset of Seizova et al., an upregulated interferon response pathway was observed in the infected cells as compared to the mock-infected cells; however, in our dataset, downregulation of this pathway was observed in the infected cells compared to the mock-infected cells. One possible explanation is the difference in the baseline upregulation level of these pathways in both infected cells and cells^low parasite reads^. The bulk RNA-Seq data simulated by totaling whole cells in the dataset showed that with a higher infection rate in the Large dataset [~20% infection which is equivalent to the previous study (Seizova et al., 2021)], upregulation of the interferon response pathways was higher than that in the Deep dataset, as indicated by the CPM-normalized STAT1 levels (597, 258, and 155 in Large dataset, Deep dataset, and mock infected culture control, respectively; **Data Set S1, Sheet 1**).

This difference in the baseline IFN response magnitude and cell number also resulted in different strengths of DEG detection (**Data Set S1, Sheets 2** and **5**). For example, the downregulation of STAT1 in the BAG1+ subset vs. cells^low parasite reads^ was detected with p-values of 6.1E−09 and 0.01 in the Large and Deep datasets, respectively. Because we used the sign of log_2_ fold change (up or down)- and p-value-based gene scoring (Van den Berge et al., 2018) for GSEA, a strong p-value in the Large dataset resulted in a more pronounced GSEA score for the IFN response pathway compared to the Deep dataset (**Supplementary Figure S4**).

Regarding the heterogeneity in host cell transcriptomes, we identified two subsets of host cells that contained BAG1+ parasites (i.e., HR-IvL and #A–#F). BAG1+ cells in the HR-IvL subset showed infection-triggered altered transcription and upregulated E2F target pathways with higher expression levels of *c-Myc*, along with a higher enrichment score for the MYC_Targets_V1 pathway. One of the major contributors to host c-Myc pathway upregulation is GRA16, which accounts for 68% of the c-Myc activation in tachyzoite-infection (Panas and Boothroyd, 2020). GRA16 is not secreted in the bradyzoite stage (Krishnamurthy and Saeij, 2018; Seizova et al., 2021); however, Mayoral et al. showed that GRA16 levels were above baseline even after 6 days of bradyzoite-infection, but were lower than those on day 1 (Mayoral et al., 2020). This residual GRA16 could contribute to upregulated c-Myc pathway in cells containing bradyzoites. However, we cannot eliminate the possibility that other effector(s) are also involved in these changes.

Despite various significant findings, the present study had a few limitations. One of these was high pH and low CO_2_ culture conditions to induce bradyzoite differentiation. The transcriptome changes observed were the changes in a stressed host cell rather normal culture conditions. Recently, Waldman et al. identified a master regulator of bradyzoite differentiation, BFD1 (Waldman et al., 2020). Conditional overexpression of BFD1 can induce bradyzoite gene profiles under non-stressed culture conditions (Waldman et al., 2020). Bradyzoites-infected cells under these non-stressed conditions would be ideal for investigating the host transcriptional changes and will reveal the general transcriptional changes associated with bradyzoite-infection.

In summary, scRNA-Seq analysis allowed us to solve the heterogeneity problem within the *in vitro* bradyzoite induction culture to detect host cell transcription alterations. In the transcriptome landscape, we identified several host pathways that were transcriptionally altered in a parasite differentiation-dependent manner. Our data provide the starting point for identification of additional parasite effectors involved in stage-dependent host transcription alterations and reveal the underlying parasite-induced heterogeneity in host cells.

## Data Availability Statement

The datasets presented in this study can be found in online repositories. The names of the repository/repositories and accession number(s) can be found in the article/**Supplementary Material**.

## Author Contributions

TS and JY contributed to conception, design and data analysis of the study. TS, TT, KH, LW, and JY contributed to interpretation of data. KT and NK contributed to the acquisition of data. TS wrote the first draft of the manuscript. All authors listed have made a substantial, direct, and intellectual contribution to the work and approved it for publication.

## Funding

This study was supported by the JSPS KAKENHI Grant Number JP17H03912 (JY) and the NIH NIAID AI134753 (LW).

## Conflict of Interest

The authors declare that the research was conducted in the absence of any commercial or financial relationships that could be construed as a potential conflict of interest.

## Publisher’s Note

All claims expressed in this article are solely those of the authors and do not necessarily represent those of their affiliated organizations, or those of the publisher, the editors and the reviewers. Any product that may be evaluated in this article, or claim that may be made by its manufacturer, is not guaranteed or endorsed by the publisher.

## Supplementary Material

Supplementary Figure S1 Additional characterization for Deep dataset **(A)** Single cell suspension used for the scRNA-seq library preparation were attached to slide glass with cytospin. Bradyzoite parasitophorous vacuole was stained with FITC-conjugated DBA lectin. Total parasites were stained with anti-Toxoplasma rabbit polyclonal antibody and host nuclei were visualized by staining DNA with DAPI. Host nuclei number and the number of parasitophorous vacuoles containing at least two parasites were counted and used to estimate infection rate. From three cytospin slides, at least 300 host nuclei were counted for each sample. Mean infection rate for the single cell suspension sample used for the scRNA-seq was 5.1% (± 1.2% SD). **(B, C)** Cell cycle and intrinsic heterogeneity drives the cell clustering in the bradyzoite infected cells. **(B)** Each cell was assigned cell cycle phase G1, G2M, or S based on the gene expression profile. Cluster assignment of #A-#F and HR-IvL were retrieved from the clustering analysis done in . UMAP visualization as done in was overlayed with cell cycle phase assignment G1, G2M, or S. **(C)** Clustering of cells using host gene expression profiles with mock control cells and parasite-exposed cells was conducted as described in . Parasite-exposed cells are labeled with the clusters (#A-#F and HR-IvL) as detected in , and mock control cells are shown as #NonEx (Non-exposed cells). Intrinsic heterogeneity separated HFF cells into two large subsets (as represented with #C, #D and #E, #F in parasite exposed cell) and G2M cell cycle-related clusters (as represented with #A and #B in parasite exposed cells).

Supplementary Figure S2 Clustering of host cells in bradyzoite induction culture with single cell transcriptome (Large dataset). Analysis of the large cell number dataset (Large dataset) is shown **(A–D)**. Analysis of batch effect-corrected merged dataset of Large and Deep datasets is shown **(E, F)**. **(A)** Single-cell level transcriptome of parasite-exposed host cells under bradyzoite induction conditions was used for the clustering of host cells with host genes. The cluster number does not correspond to those with the Deep dataset in . **(B)** Log_2_ transformed mRNA molecule counts mapped to parasites are plotted to indicate the parasite load per cell for each cluster. By counting the infected cells using immunofluorescence analysis, infection rate was estimated as 20%. The top 20% cells in the parasite-mapped mRNA counts were assigned to infected cells. The blue dotted line shows the cut-off level for separating putative infected and cells ^low parasite reads^. **(C)** Infected cells were further plotted for the expression of the canonical tachyzoite marker SAG1 and bradyzoite marker BAG1. Log_10_ values of the mRNA count + 1 for each gene are shown. The cut-off line of the row mRNA count number above three was used to assign each cell positive for SAG1/BAG1. **(D)** Parasite differentiation categories are overlaid by host cell clustering. **(E, F)** To correct the batch effect caused by different scRNA-Seq depths in the Large and Deep datasets, the Seurat pipeline using canonical correlation analysis was performed as described in the Seurat manual (https://satijalab.org/seurat/articles/merge_vignette.html). **(E)** UMAP plots for the merged scRNA-Seq dataset of the Deep and Large datasets. The cluster numbers 0–8 in the Deep and Large datasets correspond to each other. **(F)** Putative infection status estimated by parasite load in the cells was overlaid onto the UMAP clustering plot. Cluster 7 was highly enriched in the infected cells in both the Large and Deep datasets.

Supplementary Figure S3 Parasite load distributions in the four BAG1/SAG1-classified infected cell subsets. **(A)** mRNA count mapped to *Toxoplasma* genome for each infected cell are shown. BAG1+: cells with *T. gondii* ^SAG1-/BAG1+^, SAG1+: cells with *T. gondii* ^SAG1+/BAG1-^, +/+: cells with *T. gondii* ^SAG1+/BAG1+^, and -/-: cells with *T. gondii* ^SAG1-/BAG1-^. ** shows adjusted *p-value* by Tukey HSD test < 0.01. **(B)** Differentially expressed parasite genes among the four subsets were subjected to geneset enrichment analysis using tachyzoite and bradyzoite differentiation genesets, KEGG pathway, and intracellular and extracellular parasite genesets. Normalized enrichment scores for gene set enrichment analysis (GSEA) are shown. Enriched pathways for upregulated genes are shown in red and those for downregulated genes are shown in blue. Enriched pathways with FDR less than 0.05 in at least one of the comparisons are shown. Pathways with FDR < 0.05 are shown with solid circles. B: BAG1+ cells, S: SAG1+ cells, DP (double positive): +/+, DN (double negative): ND/ND.

Supplementary Figure S4 Host transcription alterations in BAG1+ subset are similar between Deep and Large dataset. The large cell number dataset (Large dataset) and Deep dataset were analyzed to see transcription alteration in SAG1/BAG1-based classification subsets compared to cells ^low parasite reads^. It should be noted that in the Large dataset, only five SAG1+ cells and three +/+ cells were detected; thus, the analysis for these subsets was not sufficiently robust. Instead of the normalized enrichment scores in , -log10 FDR values for gene set enrichment analysis (GSEA) are shown as a size of circle to cover the large variety. Enriched pathways for upregulated genes are shown in red and those for downregulated genes are shown in blue. Enriched pathways with FDR less than 0.001 in at least one comparison are shown. Pathways with an FDR < 0.001 are shown as solid circles. B: BAG1+ cells, S: SAG1+ cells, DP (double positive): +/+, DN (double negative): ND/ND.

Supplementary Figure S5 Parasite gene expression in BAG1+HR-IvL and BAG1+#A-#F subsets. **(A)** Parasitic mRNA count for BAG1+HR-IvL and BAG1+#A-#F cells are shown. Student’s *t-test p-value* is 0.21. **(B)** Normalized enrichment score (NES) and FDR values for gene set enrichment analysis (GSEA) are shown. NES > 0 means gene pathway enriched for the upregulated genes in BAG1+HR-IvL compared to BAG1+#A-#F. Enriched pathways with FDR less than 0.05 are shown with Extracellular gene set. **(C)** Counts per million reads mapped to parasite transcriptome (CPM) value for each gene per each cell were transformed to log1p-value; ln(CPM+1). Box plot to show the quantile values and violin plot to show the distribution of the values are overlayed with dot plot to show the expression value for each single cell. BAG1+HR-IvL and BAG1+#A-#F cell subsets are shown.

Supplementary Figure S6 Changes in parasitophorous vacuole number and size distribution over the six days of culturing in bradyzoite induction medium. **(A)** Infection rate and **(B)** the parasitophorous vacuole sizes were characterized on days 2, 4, and 6 of bradyzoite induction culture. Data from independent triplicate experiments are shown. Images from each replicate and each time point, namely, at least 200 host cells, were used for the analysis. No significant differences was observed in infection rate (one way ANOVA *p-value* = 0.36). *: adjusted *p-value* < 0.05, **: adjusted p-value < 0.01 with Tukey’s ‘Honest Significant Difference’ method.
