# Supplementary figures and images for: Single Cell Transcriptomes of In Vitro Bradyzoite Infected Cells Reveals Toxoplasma gondii Stage Dependent Host Cell Alterations
Source: Front Cell Infect Microbiol. 2022 Mar 14;12:848693. doi: 10.3389/fcimb.2022.848693 (PMC8964302; doi:10.3389/fcimb.2022.848693)

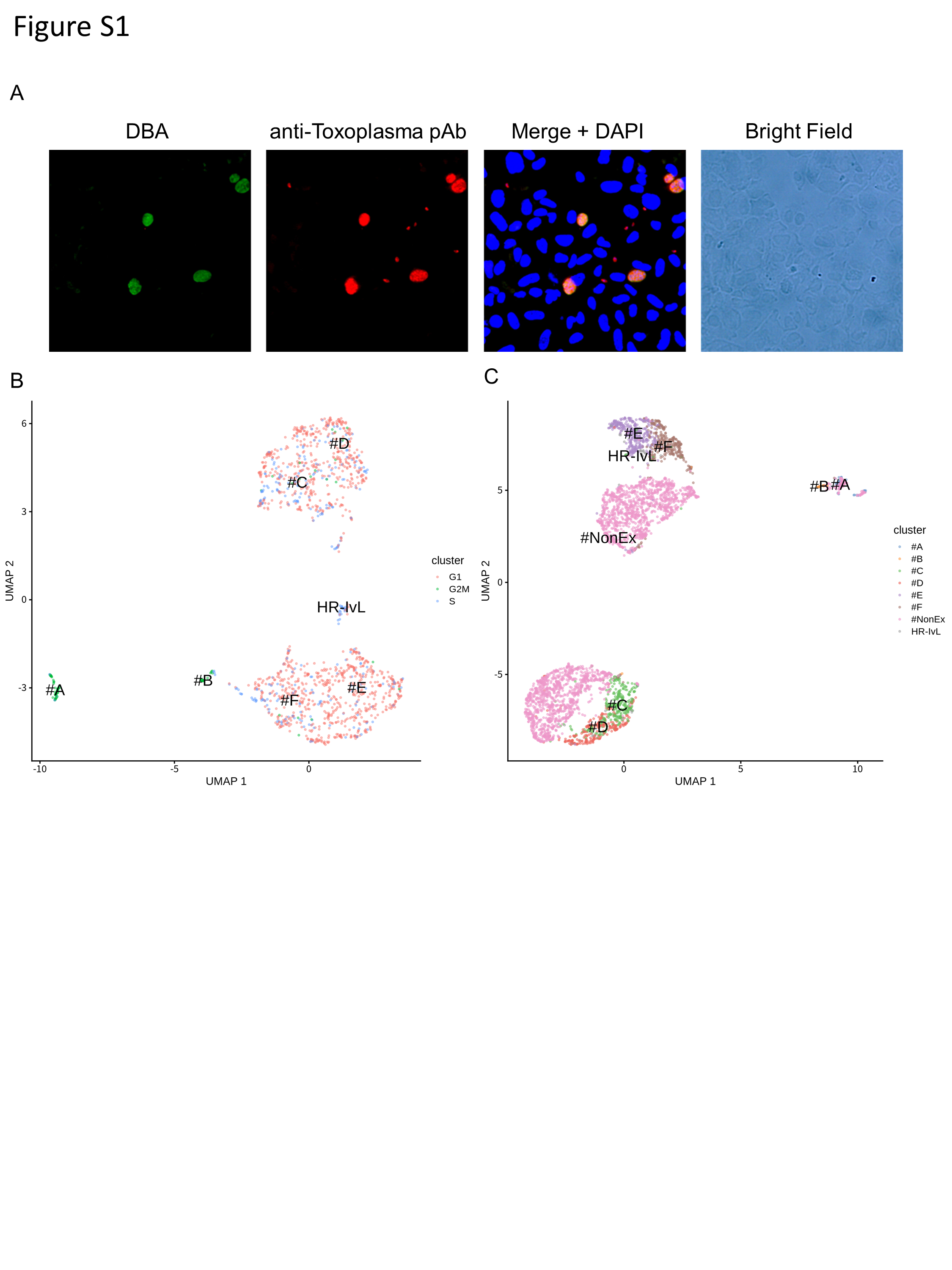

Supplement: Supplementary Figure S1 — Additional characterization for Deep dataset (A) Single cell suspension used for the scRNA-seq library preparation were attached to slide glass with cytospin. Bradyzoite parasitophorous vacuole was stained with FITC-conjugated DBA lectin. Total parasites were stained with anti-Toxoplasma rabbit polyclonal antibody and host nuclei were visualized by staining DNA with DAPI. Host nuclei number and the number of parasitophorous vacuoles containing at least two parasites were counted and used to estimate infection rate. From three cytospin slides, at least 300 host nuclei were counted for each sample. Mean infection rate for the single cell suspension sample used for the scRNA-seq was 5.1% (± 1.2% SD). (B, C) Cell cycle and intrinsic heterogeneity drives the cell clustering in the bradyzoite infected cells. (B) Each cell was assigned cell cycle phase G1, G2M, or S based on the gene expression profile. Cluster assignment of #A-#F and HR-IvL were retrieved from the clustering analysis done in . UMAP visualization as done in was overlayed with cell cycle phase assignment G1, G2M, or S. (C) Clustering of cells using host gene expression profiles with mock control cells and parasite-exposed cells was conducted as described in . Parasite-exposed cells are labeled with the clusters (#A-#F and HR-IvL) as detected in , and mock control cells are shown as #NonEx (Non-exposed cells). Intrinsic heterogeneity separated HFF cells into two large subsets (as represented with #C, #D and #E, #F in parasite exposed cell) and G2M cell cycle-related clusters (as represented with #A and #B in parasite exposed cells). [file Image_1.tif]

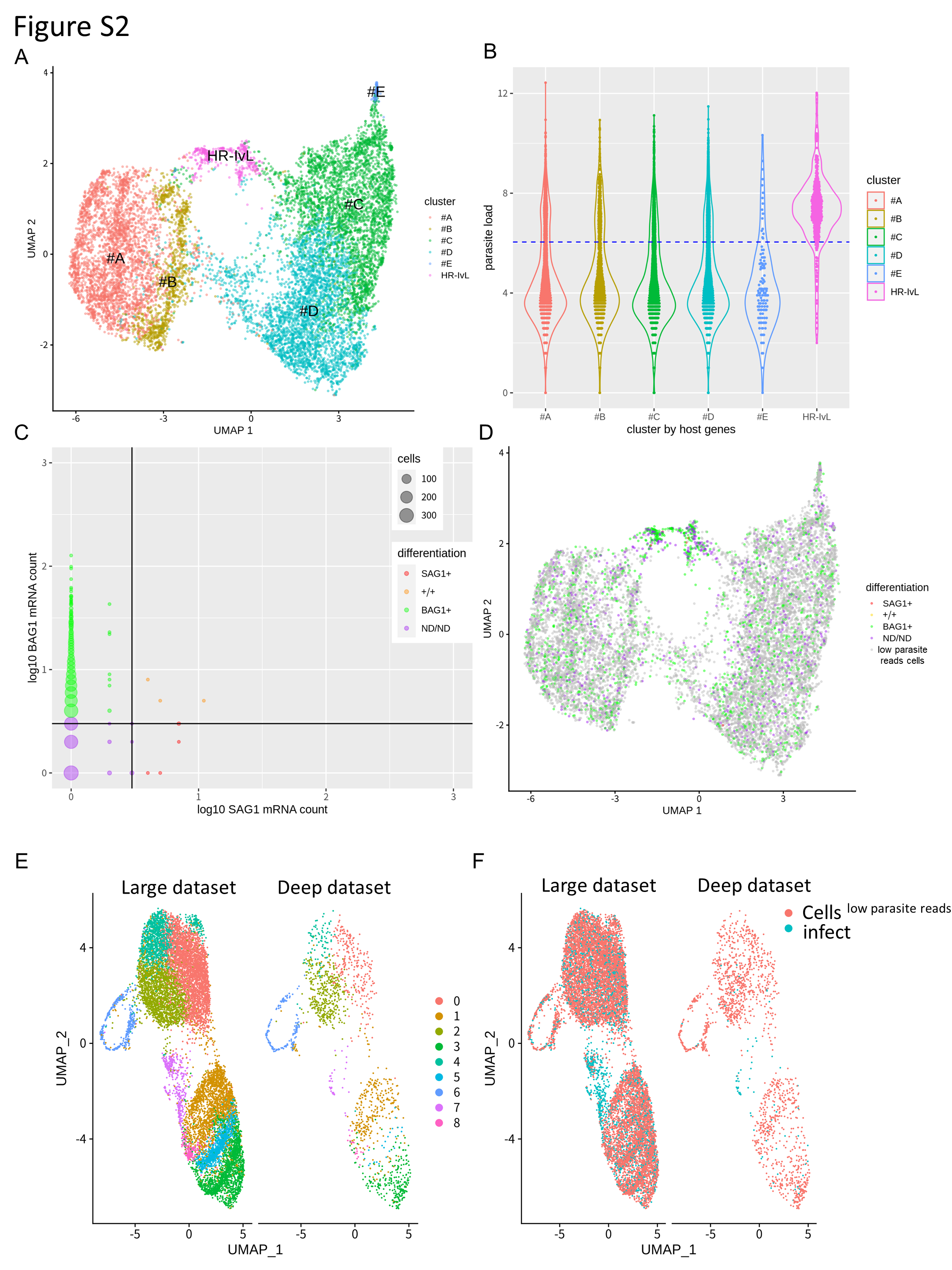

Supplement: Supplementary Figure S2 — Clustering of host cells in bradyzoite induction culture with single cell transcriptome (Large dataset). Analysis of the large cell number dataset (Large dataset) is shown (A–D). Analysis of batch effect-corrected merged dataset of Large and Deep datasets is shown (E, F). (A) Single-cell level transcriptome of parasite-exposed host cells under bradyzoite induction conditions was used for the clustering of host cells with host genes. The cluster number does not correspond to those with the Deep dataset in . (B) Log2 transformed mRNA molecule counts mapped to parasites are plotted to indicate the parasite load per cell for each cluster. By counting the infected cells using immunofluorescence analysis, infection rate was estimated as 20%. The top 20% cells in the parasite-mapped mRNA counts were assigned to infected cells. The blue dotted line shows the cut-off level for separating putative infected and cells low parasite reads. (C) Infected cells were further plotted for the expression of the canonical tachyzoite marker SAG1 and bradyzoite marker BAG1. Log10 values of the mRNA count + 1 for each gene are shown. The cut-off line of the row mRNA count number above three was used to assign each cell positive for SAG1/BAG1. (D) Parasite differentiation categories are overlaid by host cell clustering. (E, F) To correct the batch effect caused by different scRNA-Seq depths in the Large and Deep datasets, the Seurat pipeline using canonical correlation analysis was performed as described in the Seurat manual (https://satijalab.org/seurat/articles/merge_vignette.html). (E) UMAP plots for the merged scRNA-Seq dataset of the Deep and Large datasets. The cluster numbers 0–8 in the Deep and Large datasets correspond to each other. (F) Putative infection status estimated by parasite load in the cells was overlaid onto the UMAP clustering plot. Cluster 7 was highly enriched in the infected cells in both the Large and Deep datasets. [file Image_2.tif]

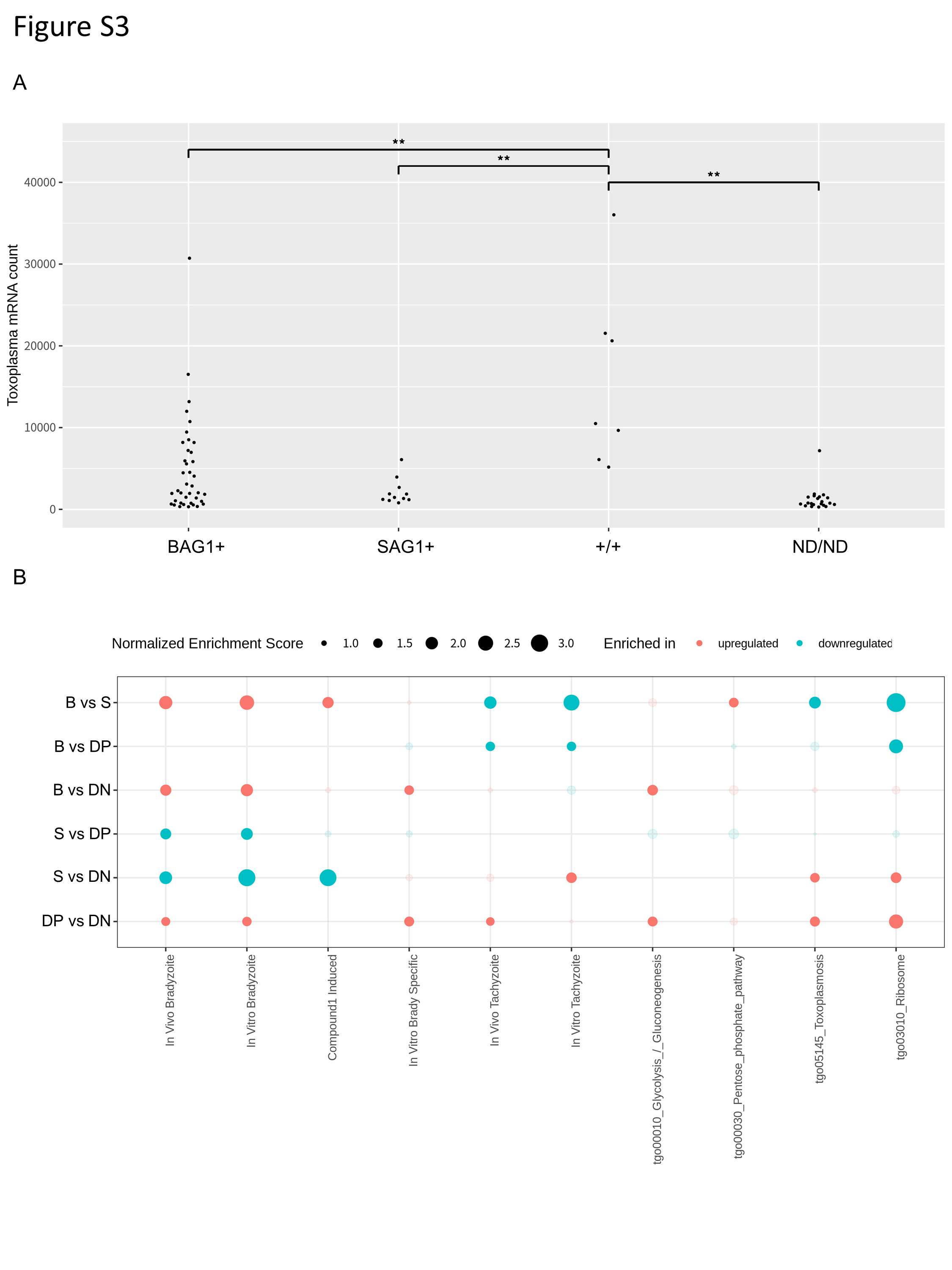

Supplement: Supplementary Figure S3 — Parasite load distributions in the four BAG1/SAG1-classified infected cell subsets. (A) mRNA count mapped to Toxoplasma genome for each infected cell are shown. BAG1+: cells with T. gondii SAG1-/BAG1+, SAG1+: cells with T. gondii SAG1+/BAG1-, +/+: cells with T. gondii SAG1+/BAG1+, and -/-: cells with T. gondii SAG1-/BAG1-. ** shows adjusted p-value by Tukey HSD test < 0.01. (B) Differentially expressed parasite genes among the four subsets were subjected to geneset enrichment analysis using tachyzoite and bradyzoite differentiation genesets, KEGG pathway, and intracellular and extracellular parasite genesets. Normalized enrichment scores for gene set enrichment analysis (GSEA) are shown. Enriched pathways for upregulated genes are shown in red and those for downregulated genes are shown in blue. Enriched pathways with FDR less than 0.05 in at least one of the comparisons are shown. Pathways with FDR < 0.05 are shown with solid circles. B: BAG1+ cells, S: SAG1+ cells, DP (double positive): +/+, DN (double negative): ND/ND. [file Image_3.tif]

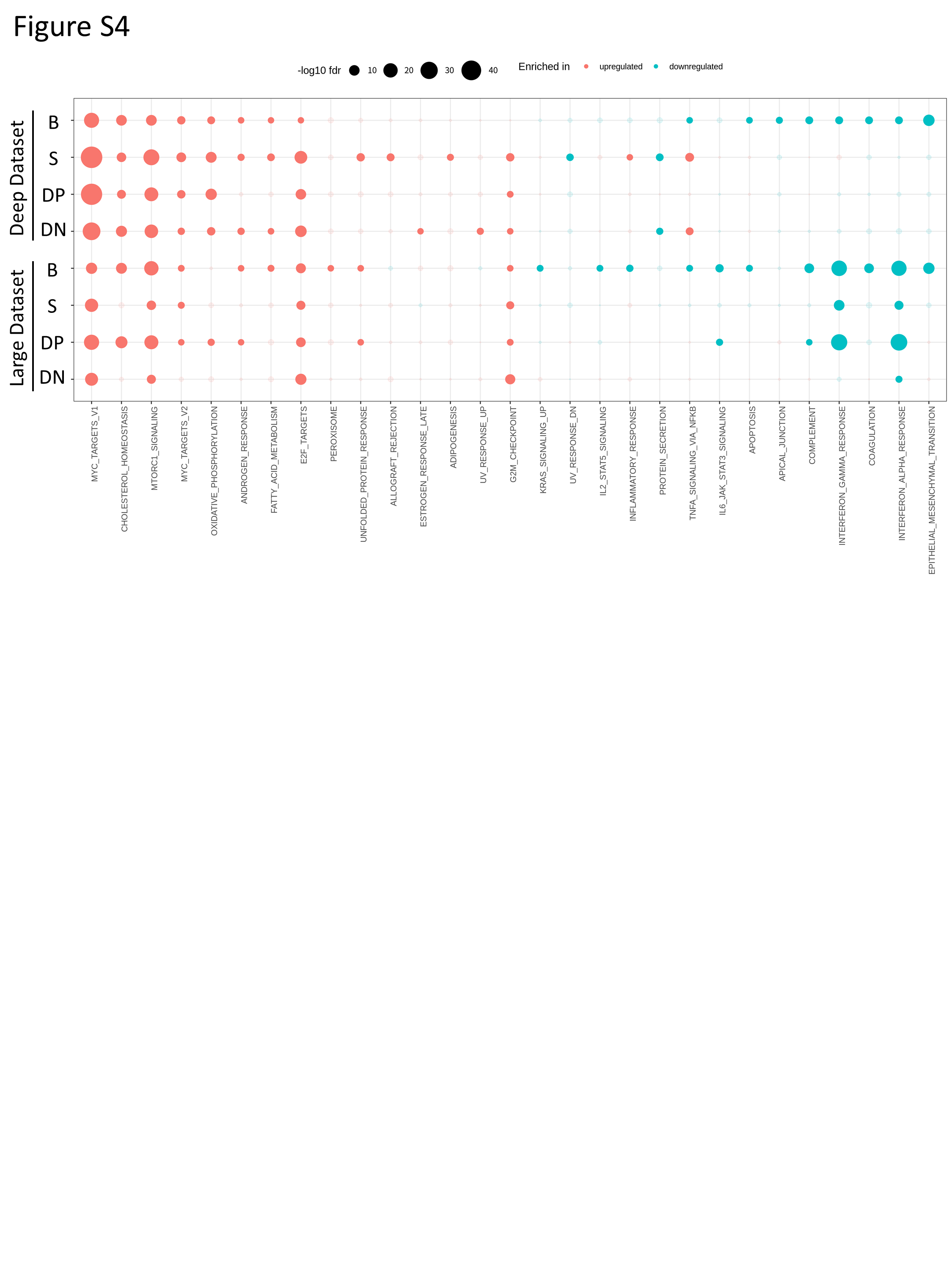

Supplement: Supplementary Figure S4 — Host transcription alterations in BAG1+ subset are similar between Deep and Large dataset. The large cell number dataset (Large dataset) and Deep dataset were analyzed to see transcription alteration in SAG1/BAG1-based classification subsets compared to cells low parasite reads. It should be noted that in the Large dataset, only five SAG1+ cells and three +/+ cells were detected; thus, the analysis for these subsets was not sufficiently robust. Instead of the normalized enrichment scores in , -log10 FDR values for gene set enrichment analysis (GSEA) are shown as a size of circle to cover the large variety. Enriched pathways for upregulated genes are shown in red and those for downregulated genes are shown in blue. Enriched pathways with FDR less than 0.001 in at least one comparison are shown. Pathways with an FDR < 0.001 are shown as solid circles. B: BAG1+ cells, S: SAG1+ cells, DP (double positive): +/+, DN (double negative): ND/ND. [file Image_4.tif]

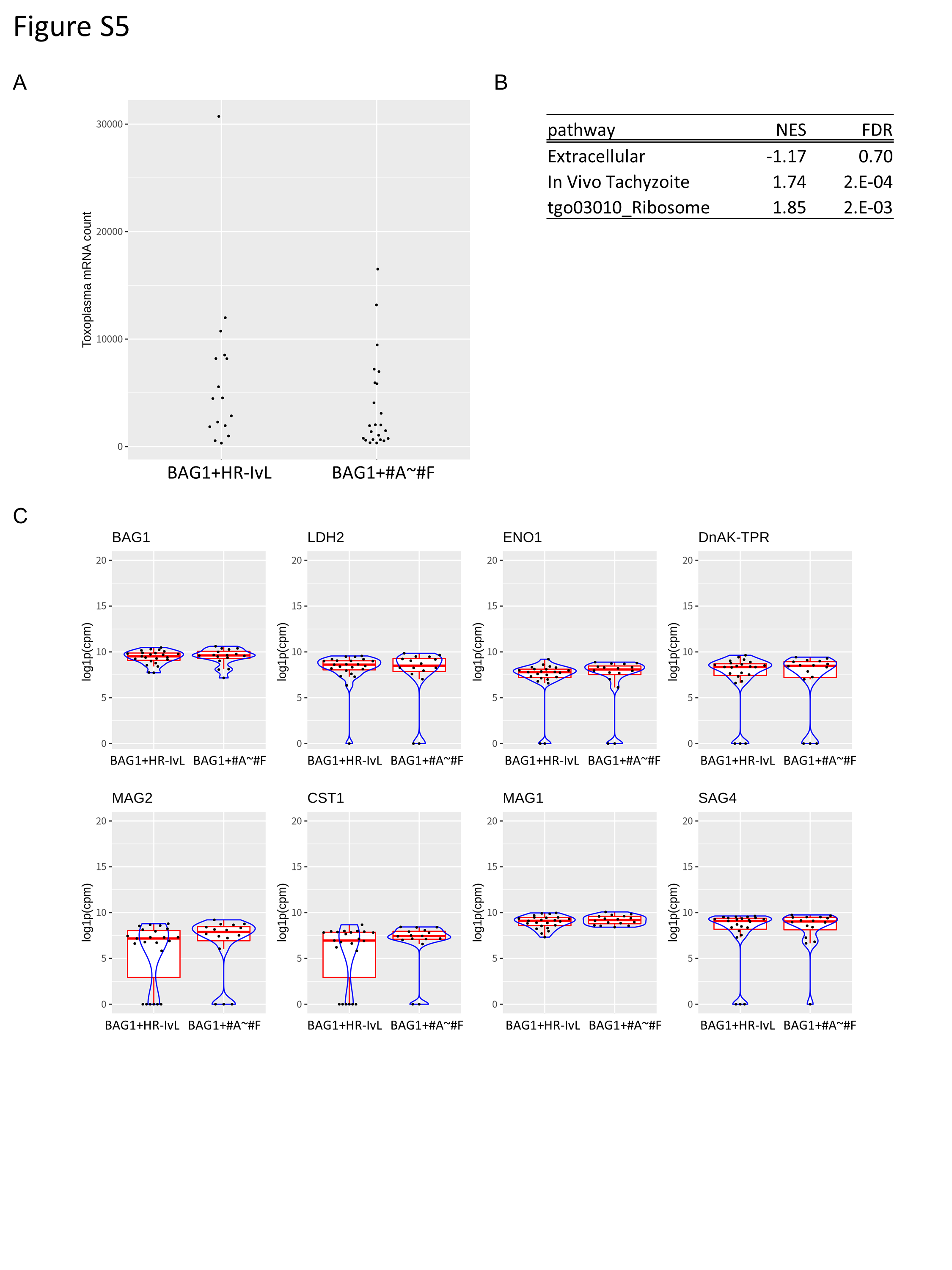

Supplement: Supplementary Figure S5 — Parasite gene expression in BAG1+HR-IvL and BAG1+#A-#F subsets. (A) Parasitic mRNA count for BAG1+HR-IvL and BAG1+#A-#F cells are shown. Student’s t-test p-value is 0.21. (B) Normalized enrichment score (NES) and FDR values for gene set enrichment analysis (GSEA) are shown. NES > 0 means gene pathway enriched for the upregulated genes in BAG1+HR-IvL compared to BAG1+#A-#F. Enriched pathways with FDR less than 0.05 are shown with Extracellular gene set. (C) Counts per million reads mapped to parasite transcriptome (CPM) value for each gene per each cell were transformed to log1p-value; ln(CPM+1). Box plot to show the quantile values and violin plot to show the distribution of the values are overlayed with dot plot to show the expression value for each single cell. BAG1+HR-IvL and BAG1+#A-#F cell subsets are shown. [file Image_5.tif]

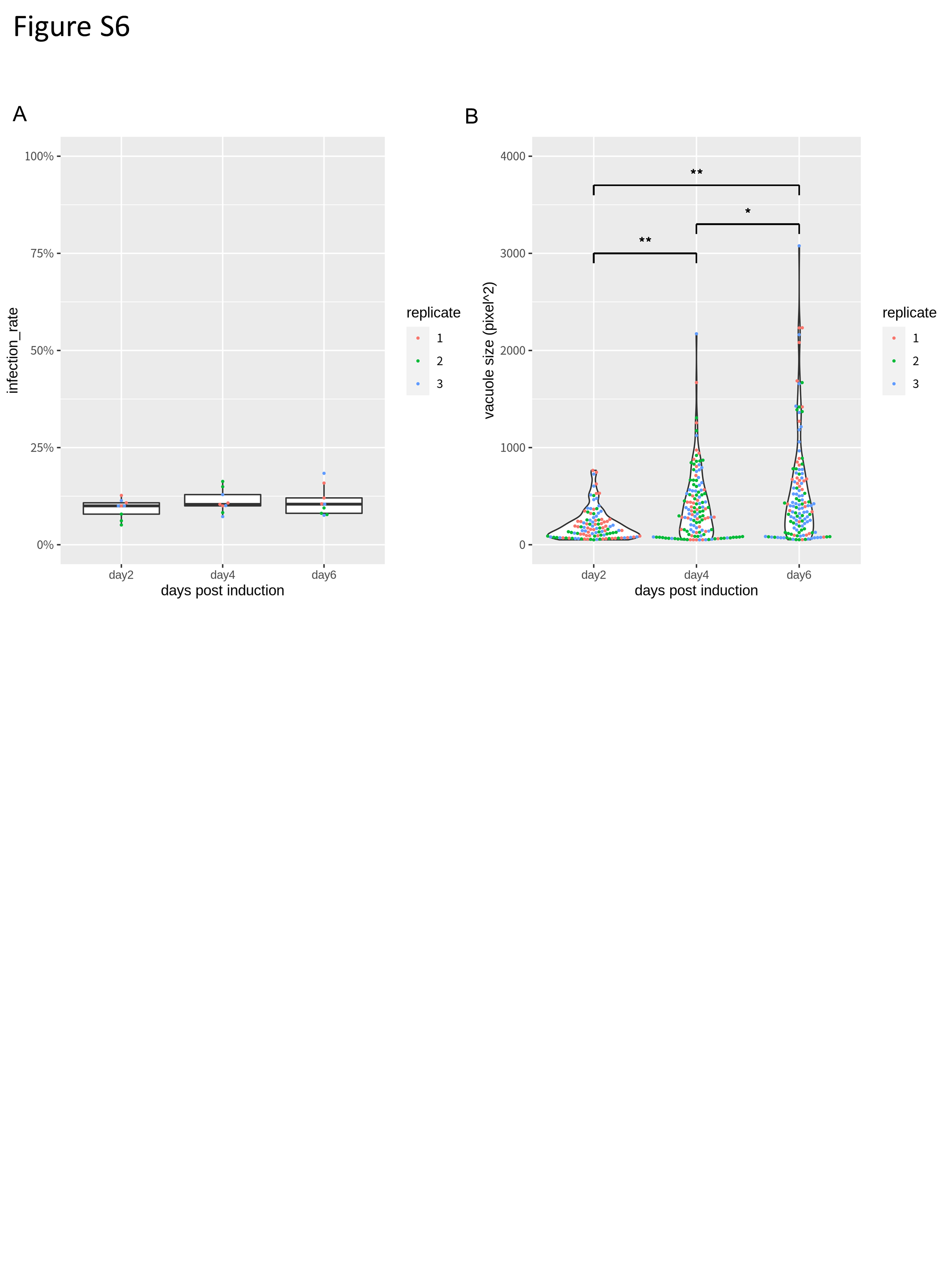

Supplement: Supplementary Figure S6 — Changes in parasitophorous vacuole number and size distribution over the six days of culturing in bradyzoite induction medium. (A) Infection rate and (B) the parasitophorous vacuole sizes were characterized on days 2, 4, and 6 of bradyzoite induction culture. Data from independent triplicate experiments are shown. Images from each replicate and each time point, namely, at least 200 host cells, were used for the analysis. No significant differences was observed in infection rate (one way ANOVA p-value = 0.36). *: adjusted p-value < 0.05, **: adjusted p-value < 0.01 with Tukey’s ‘Honest Significant Difference’ method. [file Image_6.tif]
